# Evaluation of Volatile Organic Compounds and Carbonyl Compounds Present in the Cabins of Newly Produced, Medium- and Large-Size Coaches in China

**DOI:** 10.3390/ijerph13060596

**Published:** 2016-06-15

**Authors:** Yan-Yang Lu, Yi Lin, Han Zhang, Dongxiao Ding, Xia Sun, Qiansheng Huang, Lifeng Lin, Ya-Jie Chen, Yu-Lang Chi, Sijun Dong

**Affiliations:** 1 Key Lab of Urban Environment and Health, Institute of Urban Environment, Chinese Academy of Sciences, Xiamen 361021, China; yylu@iue.ac.cn (Y.-Y.L.); ylin@iue.ac.cn (Y.L.); hzhang@iue.ac.cn (H.Z.); dxding@iue.ac.cn (D.D.); xiasun@iue.ac.cn (X.S.); qshuang@iue.ac.cn (Q.H.); lflin@iue.ac.cn (L.L.); lywchen@163.com (Y.-J.C.); ylchi@iue.ac.cn (Y.-L.C.); 2 College of Resources and Environment, University of Chinese Academy of Sciences, Beijing 100049, China; 3 Center for Excellence in Urban Atmospheric Environment, Institute of Urban Environment, Chinese Academy of Sciences, Xiamen 361021, China

**Keywords:** volatile organic compounds (VOCs), carbonyl compounds (CCs), coach, in-vehicle, temperature, humidity

## Abstract

An air-conditioned coach is an important form of transportation in modern motorized society; as a result, there is an increasing concern of in-vehicle air pollution. In this study, we aimed to identify and quantify the levels of volatile organic compounds (VOCs) and carbonyl compounds (CCs) in air samples collected from the cabins of newly produced, medium- and large-size coaches. Among the identified VOCs and CCs, toluene, ethylbenzene, xylene, formaldehyde, acetaldehyde, acrolein/acetone, and isovaleraldehyde were relatively abundant in the cabins. Time was found to affect the emissions of the contaminants in the coaches. Except for benzaldehyde, valeraldehyde and benzene, the highest in-vehicle concentrations of VOCs and CCs were observed on the 15th day after coming off the assembly line, and the concentrations exhibited an approximately inverted U-shaped pattern as a function of time. Interestingly, this study also showed that the interior temperature of the coaches significantly affected the VOCs emissions from the interior materials, whereas the levels of CCs were mainly influenced by the relative humidity within the coaches. In China, guidelines and regulations for the in-vehicle air quality assessment of the coaches have not yet been issued. The results of this study provide further understanding of the in-vehicle air quality of air-conditioned coaches and can be used in the development of both specific and general rules regarding medium- and large-size coaches.

## 1. Introduction

An air-conditioned coach is an important form of transportation for business and private activities in our modern motorized society; as a result, there are increasing concerns of vehicle-related pollution [1,2,3]. In addition to the exterior gasoline-fueled engine exhaust [4], mounting evidence indicates that interior organic pollutants are emitted from interior materials, such as plastic, paint, leather, synthetic fiber, adhesive, foam cushion, and so on [5]. A study conducted to investigate the pollution of aromatic volatile organic compounds (VOCs) of different means of transportation suggested that the levels of toluene, ethylbenzene, and xylene were significantly higher in air-conditioned buses than those in other means of roadway transportation [6]. VOCs are one of the most important categories of in-vehicle air contaminants; the most frequent VOCs include benzene, toluene, ethylbenzene, xylene, and styrene. Indeed, a recent research study in Thailand reported that the in-vehicle concentrations of benzene, toluene, ethylbenzene, and xylene were 11.7, 103.0, 11.7, and 42.8 μg/m^3^, respectively, in air-conditioned buses, which were higher than the concentrations in electric sky trains and passenger boats [7]. In addition to VOCs, several published studies suggested that carbonyl compounds (CCs), such as formaldehyde, acetaldehyde, acetone, propanal, and hexanal, also showed significant contributions to in-vehicle air pollution. For example, the results of a recent study assessed the concentrations of specific VOCs and CCs inside vehicle cabins under different vehicle driving conditions in China and indicated that the mean concentrations of formaldehyde, acetaldehyde, acetone, and acrolein were 16.43, 12.47, and 20.65 mg/m^3^ (the sum of acetone and acrolein). Moreover, this study indicated that the concentrations of these compounds inside new vehicles were higher than those inside old vehicles [8]. Notably, however, previous studies primarily focused on the measurements in small passenger cars [4,9,10,11], and there are not enough data concerning the mass concentrations of CCs in the cabins of medium- or large-size coaches.

Vehicle cabins are recognized as an important and special type of indoor environment. Currently, people spend increasing amounts of time in vehicles because of lengthy commutes, long-distance travelling, and frequent traffic jams, leading to ubiquitous exposure to in-vehicle air pollutants. Moreover, the vehicle cabin is a relatively confined space with a high amount of interior materials; as a result, the in-vehicle concentrations of some organic compounds are significantly higher than those in other indoor environments or in the corresponding ambient air [9,12]. Exposure to in-vehicle air contaminants may exacerbate allergy and asthma symptoms, or may cause nose, throat or skin irritation, coughing, headaches, or general flu-like illnesses, and can even cause cancer and neurological effects [13,14,15,16]. For example, a study conducted to evaluate the health risk of in-car benzene homologues reported that in-car benzene exposure could lead to an increased risk of cancer in taxi drivers, with the average value of cancer indices being 1.21 times higher than the unacceptable value of carcinogenic risk recommended by the United States Environmental Protection Agency (USEPA) [17].

In China, in-vehicle air pollution has received extensive attention over the past decade. A study determining the types and quantities of VOCs in both new and old cars suggested that toluene, xylene, aromatic compounds, and various C7-C12 alkanes were the primary pollutants, and the highest concentration of total VOCs was demonstrated to be up to 4.94 mg/m^3^ inside new cars [18]. A similar investigation of large numbers (802) of cars also indicated that the highest concentrations of toluene, xylene, and benzene inside cars were 81, 18, and 16 times higher, respectively, than their corresponding limit levels according to the Chinese National Indoor Air Quality Standard (GB/T18883-2002); moreover, the study suggested that newer vehicles had higher concentrations of tested pollutants than older vehicles [11]. Based on the basic understanding of in-vehicle air pollution provided by these studies, the Chinese government had issued the “Vehicle Cabin VOCs and CCs Testing Methods (HJ/T 400-2007)” as guidelines for conducting the necessary measurements of in-vehicle organic pollution. Subsequently, the Guideline of Air Quality Assessment of Passenger Cars (GB/T27630-2011) was established in 2012 to help control the levels of VOCs and CCs in the cabins of small passenger cars. However, guidelines and regulations that restrict the levels of hazardous air pollutants in medium- or/and large-size coaches have not yet been issued. A complete and accurate estimation of the concentrations of air contaminants in the cabins of coaches is a key step in the development of in-coach air quality assessment and control strategies. Accordingly, the present study is aimed at evaluating the levels of both VOCs and CCs in the cabins of newly produced, medium- and large-size, air-conditioned coaches. In addition, the effects of the time after assembly line completion, the in-vehicle temperature, and the interior relative humidity of the coaches on the levels of VOCs and CCs are also discussed in this study.

## 2. Experimental Section

### 2.1. Description of Coaches

Medium- and large-size air-conditioned coaches used in this study were domestically produced in China. The sizes of the medium- and large-coaches were 90 × 25 × 34 m^3^ and 120 × 25 × 37 m^3^, respectively. Each of the medium- and large-size coaches were equipped with a grey-black dashboard, a black leather-like steering wheel, a grey plastic floor board, a grey plastic luggage rack, and molding. However the medium-size coaches had passenger seats covered with ivory synthetic leather, whereas the large-size coaches had passenger seats covered with blue fabric. All of the coaches examined were tested within 30 days after coming off the assembly line. The plastic film covering the surface of the trim materials of the coaches was removed before sampling, and the exhaust pipe was wrapped with materials during the sampling to avoid fuel leakage.

### 2.2. Air Sampling

In-vehicle air monitoring was conducted in a specified parking garage located in a coach factory. The garage was air conditioned and well ventilated. For pretreatment, the coaches were moved into the parking garage, the engines were turned off, and the sampling system (GilAir Plus, Sensidyne, St. Petersburg, FL, USA) was configured inside the coaches’ cabins. The sampling points were evenly distributed in the central axis of the cabin, at a location of approximately 1.2 m above the floor (approximately in the breathing zone). All the doors and windows were open for 6 h. Next, the coaches were sealed for 16 h until sampling. Air samples for VOCs were collected through Tenax-TA tubes at an air flow rate of 25 mL/min for 20 min, and air samples for CCs were collected through DNPH-impregnated cartridges at an air flow rate of 400 mL/min for 30 min. The interior temperature and relative humidity were determined during sampling. Field air samples were also collected to examine the ambient air concentrations (Table 1). After sampling, the sorbent tubes were sealed with plastic or brass end caps, placed in a sealed plastic box, and then transported to the laboratory. Analyses of all samples were performed within one week.

### 2.3. Analytics

Standard solutions of fifteen carbonyl-DNPH derivatives (47285-U) were purchased from Supelco (Bellefonte, PA, USA), and a high-performance liquid chromatography (HPLC) system (Hitachi D-2000, Tokyo, Japan) coupled with a diode-array detector was used for CCs detection. Chromatographic separation was achieved using a Venusil XBP C18 column (5 µm, 250 × 4.6 mm I.D., Bonna-Agela Technologies, Tianjin, China). The mobile phase was a mixture of water-acetonitrile (40:60, *v*/*v*). Acetonitrile was purchased from Honeywell Burdick & Jackson (Muskegon, MI, USA), and the water was deionized and purified using a Milli-Q Water System. The injected sample volume was 20 µL, and the flow-rate of the mobile phase was 1.0 mL/min. The oven temperature was set at 30 °C. The detection wavelength was set at 365 nm. The limit of detection (LOD) for all identified CCs was determined by diluting solutions with a known concentration until the S/N (signal-to-noise ratios) was <3. (Table 2). The calibration linearity of the DNPH derivatives of CCs was investigated using five standard concentrations corresponding to the expected range of concentrations found in the real air samples (by adding the proper amount of 15 carbonyl-DNPH stock standard into DNPH-silica cartridges and then eluting with acetonitrile). Good linearity was observed from 0.03 to 0.3 µg/mL, and the determination coefficients (*R*^2^) were higher than 0.9930 for all the CCs (Table 3). In addition, the recoveries for all CCs were in the range of 80%–120%, with an RSD% lower than 12% (Table 4).

A mixed standard solution of seven VOCs was purchased from the Institute for Environmental Reference Materials of Ministry of Environmental Protection (IERM, Beijing, China) and was assessed using a stainless steel adsorption tube (1/4 inch × 9 cm, PerkinElmer, Norwalk, CT, USA) charged with Tenax-TA (60/80 mesh, Supelco, Bellefonte, PA, USA) by direct thermal desorption (Turbo Matrix 650 ATD, Shelton, CT, USA) and gas chromatography/mass spectrometry (GC–MS) (Shimadzu, QP2010 Plus, Kyoto, Japan). Two-stage desorption analysis mode was used. First, the collected VOCs were thermally desorbed at a split ratio of 100:1 and then refocused onto a cold trap, filled with Tenax-TA and graphitized carbon, and cooled to −30 °C; then, the cold trap was flash heated to inject all the analytes into the GC–MS unit. The analytical conditions of GC–MS were as follows: capillary column: DB-624 (Agilent, Santa Clara, CA, USA, 60 m × 0.25 mm, 1.4 μm); oven temperature: first, programmed from 38 to 42 °C at 3 °C/min and kept for 1 min, next, raised to 60 °C at 4 °C/min, from 60 to 150 °C at 8 °C/min, from 150 to 175 °C at 4 °C/min, and finally raised to 230 °C at 30 °C/min and maintained for 4 min; carrier gas: helium; flow rate: 1.66 mL/min; and interface temperature: 230 °C. The conditions for the ionization method of the MS were as follows: ionization energy: 70 eV; ion source temperature: 200 °C; analytical mode: scanning for identification (scanning range: 60–300 m/z) and selected ion monitoring (SIM) for quantification. Five calibration levels were used for masses ranging from 0.5 to 20 ng. The calibration curves showed good linearity, and *R^2^* values were higher than 0.9990 for all VOCs (Table 3). The LOD was lower than 0.01 ng for all VOCs (Table 2). The recoveries were determined by adding 0.5, 5, and 20 ng VOCs into Tenax-TA tubes, and all recovery values were higher than 91%, with an RSD% lower than 3.62% (Table 5).

### 2.4. Statistical Analysis

The data are presented as the mean ± SEM, where SEM (standard error of the mean) represents the standard deviation of the sample mean estimate of a population mean and was calculated via the sample standard deviation divided by the square root of the sample size. Statistical analyses were performed using SPSS 17.0 software. The concentrations of organic compounds during different monitoring periods, interior temperatures, and relative humidity were analyzed using one-way ANOVA, followed by Bonferroni’s *post hoc* test; the values labeled with different letters were found to be significantly different from each other at *p* < 0.05. The differences between the medium- and large-size coaches were analyzed using the unpaired *t* test. A value of *p* < 0.05 was regarded as statistically significant.

## 3. Results

### 3.1. Identification of Organic Compounds in the Cabins of Air-Conditioned Coaches

**Figure 1:**
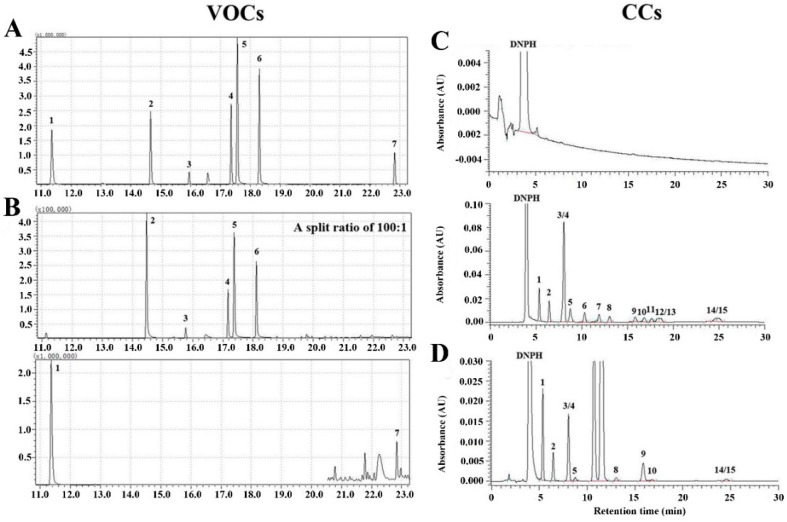
Representative chromatogram for the VOCs and CCs detected in the air-conditioned coaches. (**A**) Chromatogram for the VOCs standards spiked into Tenax TA tubes; (**B**) Typical chromatogram of VOCs in the air samples collected in the medium-size coaches. Two-stage desorption analysis mode was used. Peaks in (**A**,**B**) 1. Benzene, 2. Toluene, 3. *n*-Butyl Acetate, 4. Ethylbenzene, 5. Xylene, 6. Styrene, 7. *n*-Undecane; (**C**) HPLC chromatogram for the DNPH-derivatized CCs standards; (**D**) Typical chromatogram of CCs in the air samples collected in the medium-size air-conditioned coaches. Peaks in (**C**,**D**): 1. Formaldehyde, 2. Acetaldehyde, 3/4. Acrolein/Acetone, 5. Propionaldehyde, 6. Crotonaldehyde, 7. Butyraldehyde, 8. Benzaldehyde, 9. Isovaleraldehyde, 10. Valeraldehyde, 11. *o*-Tolualdehyde, 12. *m*-Tolualdehyde, 13. *p*-Tolualdehyde, 14/15. Hexaldehyde/2,5-Dimethylbenzaldehyde.

**Figure 2:**
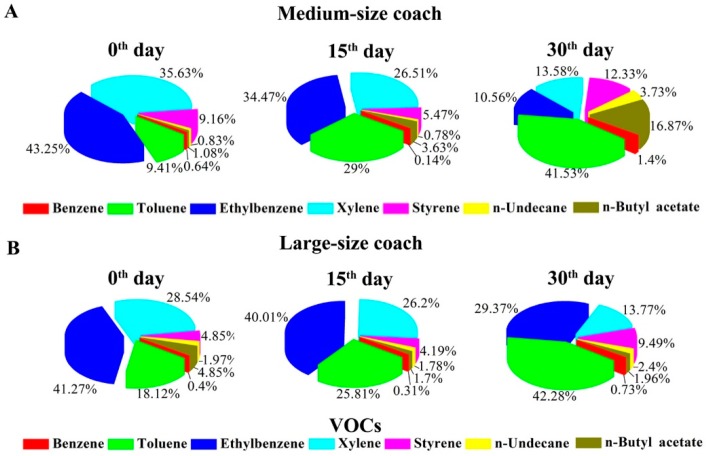

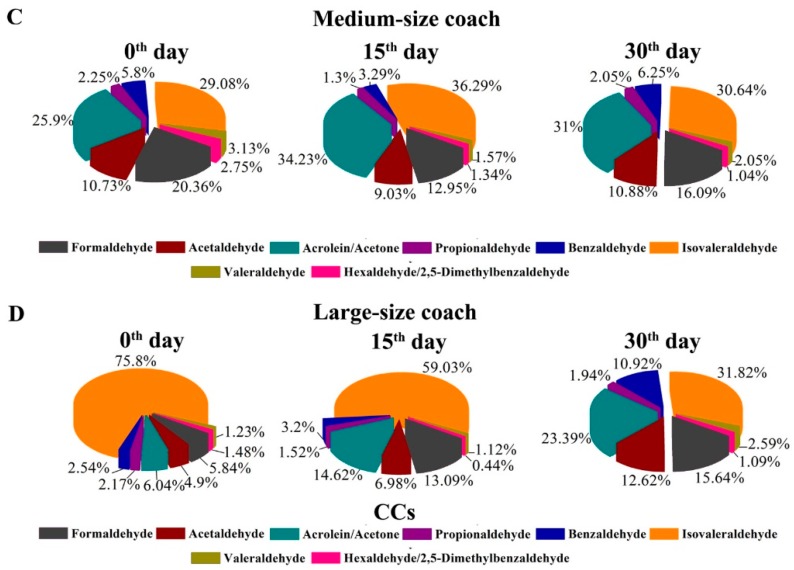
Composition ratios for in-vehicle VOCs and CCs in the collected air samples. (**A**) Composition ratios for medium-size coach VOCs; (**B**) Composition ratios for large-size coach VOCs; (**C**) Composition ratios for medium-size coach CCs. (**D**) Composition ratios for large-size coach CCs; Air samples were collected on the 0th, 15th, and 30th day after coming off the assembly line, respectively. Interior temperature: 25 ± 2 °C. Interior relative humidity: 50% ± 10%.

Seven VOCs and fifteen CCs were selected and detected in the cabins of medium- and large-size air-conditioned coaches. As shown in Figure 1 and Figure 2A,B, the most abundant VOCs present in the tested coaches were toluene, ethylbenzene, and xylene, and the concentrations of styrene and *n*-butyl acetate were higher than benzene and *n*-undecane. For CCs, Figure 1 and Figure 2C,D showed that isovaleraldehyde, acrolein/acetone, formaldehyde, and acetaldehyde had the highest concentrations in the tested coaches, and propionaldehyde, benzaldehyde, and valeraldehyde also had high emissions. Crotonaldehyde, butyraldehyde, *o*-, *m*-, and *p*-tolualdehyde were not detected in the collected air samples.

### 3.2. Emission Characteristics and the Composition Ratios of the Organic Compounds in the Cabins of Air-Conditioned Coaches

**Figure 3:**
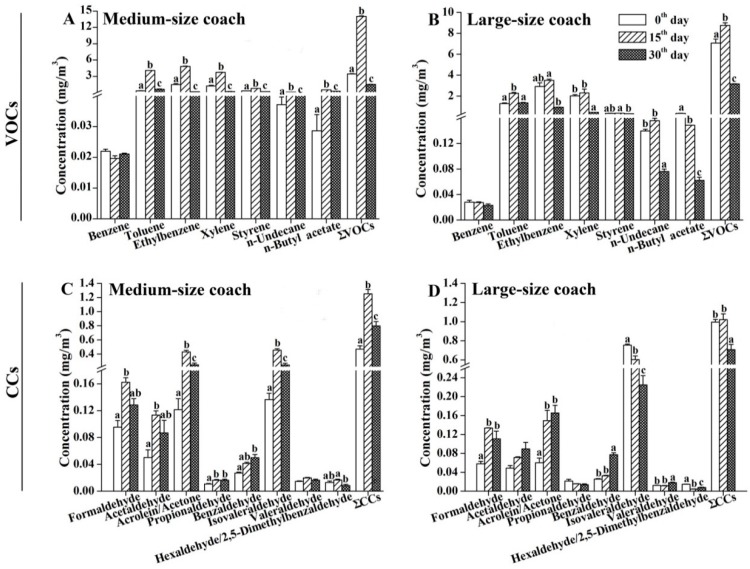
The emission amounts and characteristics of the organic compounds in the medium- and large-size air-conditioned coaches. Air samples were collected on the 0th, 15th, and 30th day after coming off the assembly line, respectively. Interior temperature: 25 ± 2 °C. Interior relative humidity: 50% ± 10%. (**A**,**B**) Levels of VOCs in the tested air samples; (**C**,**D**) Levels of CCs in the tested air samples. The results are expressed as the mean ± SEM. Values labeled with different letters are significantly different from each other at *p* < 0.05.

The levels of VOCs and CCs were measured in the air samples collected from the cabins of medium- and large-size coaches on the 0th, 15th, and 30th day after coming off the assembly line. Figure 3A shows that the concentrations of most VOCs (except for benzene) and the sum of the monitored VOCs (∑VOCs) in medium-size coaches were significantly higher for the 15th-day monitoring period than those for both the 0th- and 30th-day monitoring periods. Moreover, the emission levels of toluene, ethylbenzene, xylene, and styrene for the 15th-day monitoring period were higher than the corresponding established values of the Guideline for Air Quality Assessment of Passenger Cars (GB/T 27630-2011) in China. The lowest levels of toluene, *n*-undecane, and *n*-butyl acetate in the medium-size coaches were observed for the 0th-day monitoring period, whereas the lowest levels of ethylbenzene, xylene, styrene, and ∑VOCs were observed for the 30th-day monitoring period. The concentrations of styrene in the medium-size coaches were higher than the values of GB/T 27630-2011 throughout the monitoring period. Similar to the medium-size coaches, the emissions of most VOCs and ∑VOCs exhibited an inverted U-shaped pattern in the large-size coaches, except for the highest concentration of *n*-butyl acetate, which was observed for the 0th-day monitoring period (Figure 3B). Although the levels of ethylbenzene and xylene in the large-size coaches were higher than the values of GB/T 27630-2011 for both the 0th- and 15th-day monitoring periods, the emissions of these compounds were lower than the GB/T 27630-2011 values for the 30th-day monitoring period. For the CCs, similar emission characteristics were observed in the medium-size coaches; however, those were different in the large-size coaches. Among the eight tested CCs, the highest concentrations of propionaldehyde and benzaldehyde were observed for the 30th-day monitoring period in the medium-size coaches, whereas the other CCs and ∑CCs exhibited the highest concentrations for the 15th-day monitoring period (Figure 3C). In the large-size coaches, the levels of formaldehyde, acetaldehyde, acrolein/acetone, benzaldehyde, and valeraldehyde were gradually increased, but the other tested CCs and ∑CCs levels were decreased from the 0th to the 30th day (Figure 3D). Notably, the concentrations of formaldehyde, acetaldehyde, and acrolein/acetone were higher than the values indicated for GB/T 27630-2011 for both the 15th- and 30th-day monitoring periods for both the medium- and large-size coaches.

The composition ratios for the in-vehicle VOCs and CCs in the collected air samples were also analyzed. Figure 2A,B showed that toluene, ethylbenzene, and xylene accounted for more than 85% of the monitored VOCs in both the medium- and large-size coaches. The proportions of ethylbenzene and xylene were continuously decreased, but the proportion of toluene was increased from the 0th to 30th day after coming off the assembly line. For CCs, no significant changes in the composition ratios were observed for the medium-size coaches throughout the monitoring periods (Figure 2C). In contrast, the proportions of isovaleraldehyde in the air samples of the large-size coaches were significantly decreased from 75.8% on the 0th day to 31.9% on the 30th day, however, the ratios of formaldehyde, acetaldehyde and acrolein/acetone were increased correspondingly with time (Figure 2D).

### 3.3. Comparison of the Emission Characteristics for Organic Compounds of the Medium- and Large-Size Air-Conditioned Coaches

**Figure 4:**
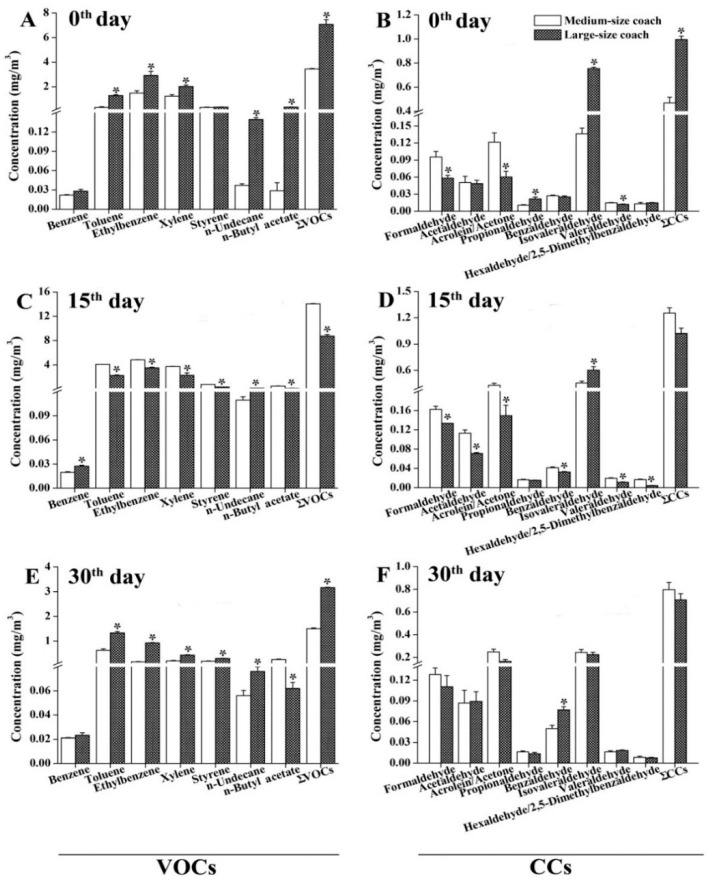
Comparison of the emission characteristics for organic compounds between the medium- and large-size air-conditioned coaches. Air samples were collected on the 0th, 15th, and 30th day after coming off the assembly line, respectively. Interior temperature: 25 ± 2 °C. Interior relative humidity: 50% ± 10%. (**A**,**C**,**E**) Levels of VOCs in the tested air samples; (**B**,**D**,**F**) Levels of CCs in the tested air samples. The results are expressed as the mean ± SEM. *: *p* < 0.05, medium-size coaches *vs.* large-size coaches.

As shown in Figure 4, we found that the concentrations of the tested organic compounds in the medium-size (41-seat) coaches differed from those in the large-size (53-seat) coaches. For the 0th-day monitoring period, the concentrations of most VOCs (except for benzene and styrene), ∑VOCs and ∑CCs were significantly higher in the large-size coaches than those in the medium-size coaches (Figure 4A,B); however, the levels of formaldehyde, acrolein/acetone, and valeraldehyde were lower in the large-size coaches than those in the medium-size coaches (Figure 4A). In contrast, for the 15th-day monitoring period, significantly higher levels for most of the tested organic compounds were observed in the medium-size coaches; benzene, *n*-undecane, and isovaleraldehyde were also at higher concentrations in the large-size coaches (Figure 4C,D). In contrast, the concentrations of most VOCs (with the exception of *n*-butyl acetate) and ∑VOCs concentrations decreased in the medium-size coaches when compared to those in the large-size coaches for 30 days after the coaches had been manufactured (Figure 4E). Almost all of the CCs had similar concentrations in both the medium- and large-size coaches for the 30-day monitoring period (Figure 4F).

### 3.4. Influence of Interior Temperature on the Emissions of Organic Compounds

**Figure 5:**
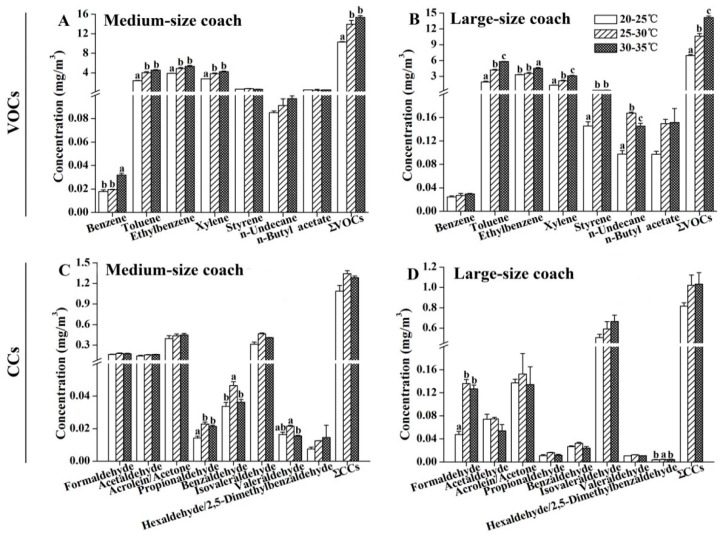
Emissions of organic compounds under different interior temperatures. (**A**,**B**) Levels of VOCs in the tested air samples; (**C**,**D**) Levels of CCs in the tested air samples. Interior relative humidity during sampling was 50% ± 10%. The results are expressed as the mean ± SEM. Values labeled with different letters are significantly different from each other at *p* < 0.05.

The relationship between the interior temperature and the concentrations of organic compounds was examined in the medium- and large-size air-conditioned coaches. As shown in Figure 5A,B, a significant positive correlation was observed between the interior temperature and the levels of ∑VOCs. The concentrations of toluene, ethylbenzene, and xylene were also significantly increased with the rise in temperature in the cabins of both the medium- and large-size coaches. In addition, the benzene emission was increased when the temperature was higher than 30 °C in the cabins of medium-size coaches (Figure 5A), and the levels of styrene and *n*-undecane in the large-size coaches were significantly elevated when the interior temperature was higher than 25 °C (Figure 5B). Unlike VOCs, no significant positive correlations were observed between the concentrations of CCs and the interior temperature for both the medium- and large-size coaches (Figure 5C,D).

### 3.5. Effects of the Interior Relative Humidity (RH) on Organic Compounds Emissions

**Figure 6:**
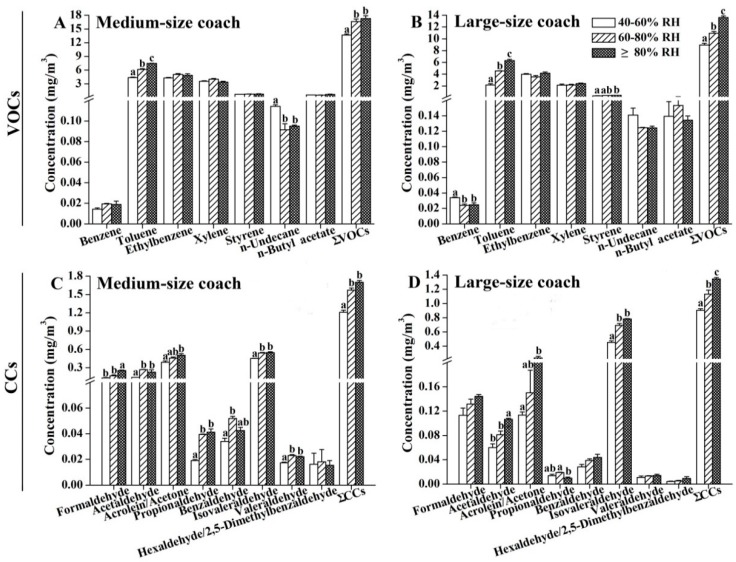
Emissions of organic compounds under different relative humidity (RH). (**A**,**B**) Levels of VOCs in the tested air samples; (**C**,**D**) Levels of CCs in the tested air samples. Interior temperature during sampling was 25 ± 2 °C. The results are expressed as the mean ± SEM. Values labeled with different letters are significantly different from each other at *p* < 0.05.

As shown in Figure 6, we found that the emissions of VOCs and CCs were also associated with the relative humidity (RH) condition of the cabins. Nevertheless, unlike the temperature effect, a significant positive correlation was observed only between RH and toluene in both the medium- and large-size air-conditioned coaches (Figure 6A,B), as well as in the ∑VOCs. All CCs, except for hexaldehyde/2,5-dimethylbenzaldehyde, had higher concentrations when the RH was higher than 60% in the medium-size coaches (Figure 6C). No significant difference was observed in the CCs levels between 60%–80% and >80% RH in the medium-size coaches (Figure 6C). For large-size coaches (Figure 6D), only concentrations of acetaldehyde, acrolein/acetone, isovaleraldehyde, and ∑CCs tended to be higher with an increasing interior RH, whereas the levels of the other tested CCs were not RH-dependent.

## 4. Discussion

Air pollution is a well-known public health risk and is considered an important contributor to the global disease burden [19]. Although the potential effects of air pollution have generally been assigned to exposure in residential locations, more studies have demonstrated that in-vehicle exposure to several air pollutants (such as VOCs and CCs) have some adverse impacts on drivers and passengers during their daily commutes. Yoshida [10] detected a total of 162 organic compounds (aliphatic hydrocarbons and aromatic hydrocarbons), and the data showed that higher concentrations of C9–C13 alkanes, ethylbenzene, and xylene were detected in the interior air of a new car. In addition, this study suggested that CCs, except for formaldehyde, appeared to be unimportant as interior contaminants. Similarly, You [18] found that toluene, xylene, some aromatic compounds, and various C7–C12 alkanes were the predominant VOCs in new vehicles, and Geiss [9] reported that aromatic compounds (e.g., benzene, toluene, ethylbenzene, and isomers of xylene), dodecane, and low molecular weight CCs (e.g., formaldehyde, acetaldehyde, and acetone) were the most abundant organic compounds present in the cabin air of used private cars. Considering that there are not enough data concerning the concentration and composition of CCs in air-conditioned coaches, our study identified and quantified fifteen CCs and seven typical VOCs potentially present in the cabins of medium- and large-size air-conditioned coaches. In accordance with previous results, we confirmed that toluene, ethylbenzene, xylene, formaldehyde, acetaldehyde, acrolein/acetone, and isovaleraldehyde were relatively abundant in the cabins of the tested coaches. In contrast, the emissions of benzene were found to be relatively lower in the tested coaches throughout the monitoring period, ranging from 19.7 to 28.0 µg/m^3^. Among the tested VOCs, benzene has been classified as a well-known carcinogen (Group 1) by the International Agency for Research on Cancer (IARC) [20], and no safe level of exposure has been recommended [21]. Therefore, adequate attention should be paid to in-vehicle benzene pollution, and further study is required to assess the relationship between the exposure to air pollutants in the air-conditioned coaches and the related health risks for drivers and passengers.

In-vehicle air contaminant levels can be influenced by the mode of transportation. Lau first reported that toluene, ethylbenzene, and xylene levels were significantly higher in air-conditioned buses than those in other forms of roadway transportation, e.g., trams, taxis, non-air-conditioned buses [6]. Mounting research results indicate that the airborne pollution in the cabins of coaches is an important issue. A recent study that surveyed the interior air pollutants in the public buses of Changsha, China, showed that the maximum concentrations of benzene, toluene, ethylbenzene, and xylenes were 106.4, 266.0, 95.9, and 234.8 μg/m^3^, respectively, in the cabins of buses [22]. Moreover, according to the analysis of multiple linear regression equations, this study suggested that bus age and interior temperature were the two most important factors influencing the in-vehicle concentrations of VOCs. Another air pollution survey of ten selected monoaromatic hydrocarbons conducted in Hangzhou, China, reported that the mean concentration of these organic compounds inside buses was 95.9 μg/m^3^, and suggested that the dominant source was vehicle emissions. Importantly, this study indicated that the mean lifetime carcinogenic risks of monoaromatic hydrocarbons for passengers and drivers were above the limit set by the USEPA [23]. Similarly, a study examining the concentration of VOCs in the public buses of Pamplona, Northern Spain, also confirmed traffic (such as driving routes and commuting periods) as the main emission source for some VOCs, such as benzene, toluene, ethylbenzene, and xylene [12]. In addition, the in-vehicle carbonyls concentrations were found to be closely associated with the vehicular service years and the fuel used in public buses [24]. Although the above-described studies mainly concentrated on the chemicals originating from the vehicular exhaust emission and the infiltration of outdoor air, interior decorations were one of the dominant sources of VOCs and CCs in the cabins of buses, especially in the newly produced buses. However, it is suggested that not much work has been performed regarding new large vehicles. To address this gap in knowledge, our study identified and quantified the levels of VOCs and CCs in air samples collected from the cabins of newly produced, medium- and large-size coaches.

In a used vehicle, the concentrations of some organic compounds may increase due to the combustion of fuel and tail pipe emissions; however, for a newly produced vehicle, the air contaminants primarily originated from the outgassing of interior materials, including plastic, paint, leather, synthetic fiber, adhesive, and foam cushion. Although many materials are subjected to emission tests by the producers before use and the results are often acceptable, the number and variety of interior materials used together in the cabin can still cause significant and undesirable impacts on the in-vehicle air quality. Faber [1] examined the air composition in the cabins of new cars of the same model, but comprised of different interior materials, and found that the total VOCs concentration ranged from 1.5 to 2.1 mg/m^3^ in the tested vehicles and that approximately 200 different organic compounds were detected in each vehicle cabin. Brodzik *et al.* [25] also investigated the air composition in nine new cars of the same model but with different interior materials; their study also indicated that the different materials used to equip a vehicle’s cabin obviously affected the type of the organic compounds emissions. Our study focused on the interior air quality of newly produced vehicles in a static condition. During the 0th-day monitoring period, we found that the mean concentrations of toluene, ethylbenzene, and xylene were 0.32, 1.49, and 1.22 mg/m^3^, respectively, in the medium-size coaches and were 1.63, 2.45, and 1.58 mg/m^3^, respectively, in the large-size coaches; these results were significantly higher than the data of small passenger cars measured within one week after manufacture [1,26]. The main reason for these differences might be the significantly larger numbers of materials used for the interior decorations and furniture in the coaches. The sizes of the medium- and large-coaches were 90 × 25 × 34 m^3^ and 120 × 25 × 37 m^3^, respectively. A larger interior surface area corresponds to an increasing number of interior materials, possibly resulting in the emission of more pollutants. In addition, the differences in interior equipment also affected the concentrations of the organic compounds. For example, the large numbers of materials found in the seats of coaches, such as poly-urethane (PU) foam, poly-propylene (PP) plastic, poly-vinyl chloride (PVC) plastic, acrylonitrile butadiene styrene (ABS) resin, and synthetic leather, emit high levels of toluene, ethylbenzene, and xylene [27]. A research report indicated that cars with a fabric trim generally had higher VOCs levels than those with a leather interior [28]. Hence, a large number of fabric seats, curtains, and plastic floor boards equipped in the tested large-size coaches might account (at least in part) for the relatively higher levels of VOCs emissions in the cabins of coaches than those in the cabins of small cars. Moreover, all of these factors might also help to explain the higher levels of most VOCs detected in the large-size coaches with fabric seats than those in the medium-size coaches with synthetic leather seats. The abundant usage of interior trims, such as roof linings, surface coatings, TV moldings, paints, adhesives, sealants, and lubricants (for the seat mechanism), may also result in the emission of VOCs. The adhesives, which were used to attach various interior parts, were found to be one of the major sources of VOCs; our findings suggest that the extensive use of adhesives, also partially explains why the levels of VOCs found in the cabin of a vehicle were higher but were undetected in the emission tests of interior parts before use. Regarding CCs, our findings suggest that particle board or plywood furniture containing formaldehyde-based resins, paints, or leather and adhesives with formaldehyde used for plastic surfaces, were the most important interior sources. Furthermore, considering that leather is known for its particularly high levels of CCs emissions, we speculate that the higher levels of CCs detected in the medium-size coaches might be merely due to the use of passenger seats covered with synthetic leather. Alternatively, we speculate that the usage of interior parts with different manufacturing dates could also result in different in-vehicle organic compounds concentrations. Further research studies should be performed to confirm the exact sources of the various organic pollutants present in the cabins of coaches.

It is generally accepted that the in-vehicle concentrations of organic compounds decrease significantly as time goes on. A used vehicle will have lower levels of air contaminants than a new one because of the ventilation of the interior after manufacturing. Yoshida [10] reported that the total concentrations of in-vehicle VOCs were approximately 14 mg/m^3^ on the day of delivery and that the levels of most compounds decreased with time (over 3 years after delivery). Recently, Faber [26] also reported that the concentrations of benzene, toluene, *o*-xylene, and *m*/*p*-xylene in the new cars tested were 11.8, 82.7, 21.2, and 89.5 μg/m^3^, respectively, and these values were approximately ten times higher than those in the used vehicles tested. Moreover, this study indicated that the concentrations of toluene and isomers of xylene increased with the increasing mileage, but they did not exceed the initial concentrations. In our study, the same coaches were tested at different times after manufacture; we found that the in-vehicle concentrations of most organic compounds were significantly higher during the 15th-day monitoring period than those detected for both the 0th- and 30th-day monitoring periods. These results suggested that the emission of organic pollutants from the interior materials into the air of the cabins of the tested coaches would take a certain amount of time and that these contaminants would accumulated in the cabins for a period of time. In addition, the results indicate that the discharge of organic pollutants from the interior materials in the tested coaches undergoes two major phases: a rapid release period and a relatively steady stage. From the 0th- to the 15th-day, a large amount of organic contaminants were quickly emitted from a wide range of interior materials, thereby significantly increasing the in-vehicle concentrations. Subsequently, a “steady state” might be achieved. Moreover, 6 h of air exchange before sampling could further reduce the in-vehicle organic compounds to lower levels. All of these factors explain the approximately inverted U-shaped emission pattern for most VOCs and CCs (toluene, ethylbenzene, xylene, styrene, formaldehyde, acetaldehyde, acrolein/acetone, and isovaleraldehyde) observed in the tested coaches. Thus, we recommend that consumers should purchase air-conditioned coaches from manufacturers after the coaches have been off the assembly line for a certain time to ensure that the air pollutants of the interior materials have been volatilized as much as possible. Notably, however, despite the ability of such measures to reduce the concentrations of several in-vehicle organic compounds to lower levels, complete elimination of the emissions of the organic compounds might be impossible. Alternatively, technologies used to purify the air, such as adsorption and catalytic elimination of air contaminants, could be used in the vehicular interior environment to decrease or prevent adverse health risks.

The interior temperature appears to be an especially important factor influencing VOCs emission [29,30], because the increased temperature inside a car cabin may promote increased vaporization and outgassing of various volatile compounds from the interior materials. Indeed, we found that the concentrations of some organic compounds were increased between 20 °C and 35 °C, but the main compositions of the air pollutants present in the cabins of the tested coaches did not change at each temperature considered. The most significant concentration growth with increasing interior temperature was observed for toluene, ethylbenzene, and xylene. Total VOCs concentrations in the air sampled from the cabins of the tested coaches were also increased with an increasing interior temperature. During summer days, the temperature in the vehicle cabin may exceed 65 °C [31]; such a high temperature would encourage a substantial emission of VOCs from the interior materials. The use of air conditioning could decrease the interior temperature and reduce the emission of some organic compounds from the interior materials; however, interference from ambient air intrusion is also possible. Indeed, a previous study comparing the levels of benzene, toluene, ethylbenzene, and xylene in air-conditioned and non-air-conditioned buses showed that the levels of these tested compounds in air-conditioned buses were 59.3%, 59.1%, 60.1%, and 60% greater than those in non-air-conditioned ones, respectively [22]. In contrast, another study examined the exposure levels of traffic-related VOCs in four popular public commuting modes and showed that there was no significant difference between air-conditioned buses (13.5 μg/m^3^) and non-air-conditioned buses (11.3 μg/m^3^) [32]. Accordingly, the effect of air conditioning on interior air pollution warrants further investigation; we recommend that the natural ventilation of air-conditioned coaches must be further increased for an appropriate period while the coaches are running. Interior relative humidity (RH) is another important factor that influences in-vehicle air quality. Several CCs, such as formaldehyde, acetaldehyde, acrolein/acetone, and isovaleraldehyde, were found to exhibit higher concentrations when the interior RH was greater than 60%. However, unlike the temperature effect, a significant positive correlation was observed between RH and the concentrations of toluene in the cabins of the tested air-conditioned coaches. As noted above, we recommend that the interior temperature and RH should be controlled at appropriate levels, especially when a new air-conditioned coach is running.

In 2012, the Guideline of Air Quality Assessment of Passenger cars (GB/T 27630-2011) was issued in China, which limited the concentrations of benzene, toluene, ethylbenzene, xylene, styrene, formaldehyde, acetaldehyde, and propionaldehyde in the cabins of newly produced cars. However, our study showed that the sum of target VOCs was observed at an average concentration of 6.32 mg/m^3^, with a minimum of 1.50 mg/m^3^ and a maximum of 14.03 mg/m^3^; moreover, the emissions of all tested VOCs, except benzene, during the 15th-day monitoring period, exceeded the established values of the GB/T 27630-2011 Guidelines. Although the levels of the identified CCs were significantly lower than those of the VOCs, the emissions of formaldehyde, acetaldehyde, and acrolein/acetone exceeded the GB/T 27630-2011 Guideline values during both the 15th- and 30th-day monitoring periods. All of these results implied that the GB/T 27630-2011 Guidelines could not be applied to evaluate the air quality in air-conditioned coaches. The data from our study will be helpful to further understand the air quality in the cabins of coaches and could contribute to the development of both specific and general rules to apply to medium- and large-size coaches; such rules have not been enforced to date, despite an evident need for them.

## 5. Conclusions

In summary, this study identified and quantified 15 CCs and seven VOCs. The data showed that toluene, ethylbenzene, xylene, formaldehyde, acetaldehyde, acrolein/acetone, and isovaleraldehyde were relatively abundant air pollutants present in the cabins of newly produced, medium- and large-size air-conditioned coaches. Except for benzaldehyde, valeraldehyde, and benzene, in-vehicle emissions of VOCs and CCs were found to exhibit an approximately inverted U-shaped pattern, and the highest in-vehicle concentrations were observed on the 15th day after coming off the assembly line. Interestingly, this study also found that the interior temperature significantly affected the VOCs emissions from the interior materials, whereas the levels of CCs were mostly influenced by the interior relative humidity of the coaches.

## Figures and Tables

**Table 1:** Average concentrations of target organic compounds in field blank sample during monitoring period.

CCs	Concentration (μg/m^3^)	VOCs	Concentration (μg/m^3^)
Formaldehyde	0.52 ± 0.04	Benzene	0.30 ± 0.02
Acetaldehyde	9.07 ± 0.75	Toluene	15.62 ± 1.31
Acrolein/Acetone	6.24 ± 0.59	*n*-Butyl Acetate	2.77 ± 0.11
Valeraldehyde	0.11 ± 0.01	Ethylbenzene	37.00 ± 3.68
*p*-Tolualdehyde	3.90 ± 0.23	Xylene	28.70 ± 1.94
Hexaldehyde/2,5-Dimethylbenzaldehyde	0.91 ± 0.06	Styrene	0.27 ± 0.01
		*n*-Undecane	2.64 ± 0.25

Data are expressed as the mean ± SEM. CCs: carbonyl compounds; VOCs: volatile organic compounds.

**Table 2:** The limit of detection (LOD) for VOCs and CCs.

CCs	LOD (μg/mL)	VOCs	LOD (ng)
Formaldehyde	0.0010	Benzene	0.00025
Acetaldehyde	0.0010	Toluene	0.00025
Acrolein/Acetone	0.0010	*n*-Butyl Acetate	0.01000
Propionaldehyde	0.0010	Ethylbenzene	0.00025
Crotonaldehyde	0.0010	Xylene	0.00025
Butyraldehyde	0.0025	Styrene	0.00250
Benzaldehyde	0.0025	*n*-Undecane	0.00025
Isovaleraldehyde	0.0050		
Valeraldehyde	0.1000		
*o*-Tolualdehyde	0.0050		
*m*-Tolualdehyde	0.0050		
*p*-Tolualdehyde	0.0050		
Hexaldehyde/2,5-Dimethylbenzaldehyde	0.0050		

**Table 3:** The linearity data for VOCs and CCs.

CCs	Regression Equation	*R* ^2^	VOCs	Regression Equation	*R*^2^
Formaldehyde	*y* = 298,120*x* + 3889	0.9966	Benzene	*y* = 473,090*x* + 2,115,074	0.9990
Acetaldehyde	*y* = 232,917*x* + 1986	0.9971	Toluene	*y* = 422,121*x* + 361,113	0.9991
Acrolein/Acetone	*y* = 328,913*x* + 3667	0.9990	*n*-Butyl Acetate	*y* = 60,706*x* + 3877	0.9991
Propionaldehyde	*y* = 246,737*x* − 116	0.9993	Ethylbenzene	*y* = 459,295*x* + 112,314	0.9992
Crotonaldehyde	*y* = 133,256*x* + 1547	0.9945	Xylene	*y* = 727,292*x* + 171,984	0.9993
Butyraldehyde	*y* = 148,648*x* − 691	0.9934	Styrene	*y* = 353,599*x* + 36,444	0.9991
Benzaldehyde	*y* = 99,102*x* + 1051	0.9951	*n*-Undecane	*y* = 177,874*x* + 103,003	0.9998
Isovaleraldehyde	*y* = 99,675*x* + 500	0.9968			
Valeraldehyde	*y* = 95,598*x* − 274	0.9965			
*o*-Tolualdehyde	*y* = 65,648*x* + 1133	0.9980			
*m*-Tolualdehyde	*y* = 79,633*x* + 259	0.9971			
*p*-Tolualdehyde	*y* = 72,383*x* + 812	0.9981			
Hexaldehyde/2,5-Dimethylbenzaldehyde	*y* = 165,117*x* + 1160	0.9906			

**Table 4:** The recovery for CCs.

CCs	0.15 μg		0.5 μg		1.5 μg	
	Recovery (%)	RSD (%)	Recovery (%)	RSD (%)	Recovery (%)	RSD (%)
Formaldehyde	108.21	4.61	114.23	6.77	116.75	9.96
Acetaldehyde	102.78	10.79	104.79	6.75	105.87	5.33
Acrolein/Acetone	98.37	7.58	96.82	9.76	117.91	3.45
Propionaldehyde	107.63	7.89	103.05	6.99	114.09	4.98
Crotonaldehyde	89.63	9.03	100.70	6.57	105.67	4.11
Butyraldehyde	91.14	9.22	98.91	8.94	99.95	5.91
Benzaldehyde	92.73	9.20	97.54	7.76	99.74	4.41
Isovaleraldehyde	98.46	5.99	118.53	7.72	114.01	3.42
Valeraldehyde	106.02	6.35	109.50	7.11	125.16	8.71
*o*-Tolualdehyde	89.75	4.90	83.30	2.12	91.31	5.52
*m*-Tolualdehyde	95.09	9.85	84.72	12.03	98.47	8.15
*p*-Tolualdehyde	82.41	10.94	81.43	8.34	90.37	2.60
Hexaldehyde/2,5-Dimethylbenzaldehyde	104.15	10.10	101.58	10.01	106.45	5.75

**Table 5:** The recovery for VOCs.

VOCs	0.5 ng		5 ng		20 ng	
	Recovery (%)	RSD (%)	Recovery (%)	RSD (%)	Recovery (%)	RSD (%)
Benzene	98.25	3.62	101.99	1.27	106.18	0.85
Toluene	105.43	3.04	96.74	1.21	99.53	2.77
*n*-Butyl Acetate	103.74	4.41	96.48	2.48	100.02	0.79
Ethylbenzene	111.22	3.61	97.07	2.42	98.59	1.54
Xylene	109.27	1.18	97.54	2.95	98.40	1.26
Styrene	106.31	2.68	96.48	2.74	96.64	1.88
*n*-Undecane	103.07	3.50	97.13	1.06	98.11	0.94

## Abbreviations

The following abbreviations are used in this manuscript:

VOCs	volatile organic compounds
CCs	carbonyl compounds
∑VOCs	the sum of monitored VOCs
∑CCs	the sum of monitored CCs
RH	relative humidity
LOD	Limit of detection
S/N	signal-to-noise ratios
